# Knee-Loading Predictions with Neural Networks Improve Finite Element Modeling Classifications of Knee Osteoarthritis: Data from the Osteoarthritis Initiative

**DOI:** 10.1007/s10439-024-03549-2

**Published:** 2024-06-06

**Authors:** Alexander Paz, Jere Lavikainen, Mikael J. Turunen, José J. García, Rami K. Korhonen, Mika E. Mononen

**Affiliations:** 1 Department of Technical Physics, University of Eastern Finland, Yliopistonranta 1, 70211 Kuopio, Finland; 2 Escuela de Ingeniería Civil y Geomática, Universidad del Valle, Cali, Colombia; 3 Science Service Center, Kuopio University Hospital, Wellbeing Services County of North Savo, Kuopio, Finland; 4 Diagnostic Imaging Center, Wellbeing Services County of North Savo, Kuopio, Finland

**Keywords:** Knee osteoarthritis, Cartilage, Finite element modeling, Neural networks

## Abstract

Physics-based modeling methods have the potential to investigate the mechanical factors associated with knee osteoarthritis (OA) and predict the future radiographic condition of the joint. However, it remains unclear what level of detail is optimal in these methods to achieve accurate prediction results in cohort studies. In this work, we extended a template-based finite element (FE) method to include the lateral and medial compartments of the tibiofemoral joint and simulated the mechanical responses of 97 knees under three conditions of gait loading. Furthermore, the effects of variations in cartilage thickness and failure equation on predicted cartilage degeneration were investigated. Our results showed that using neural network-based estimations of peak knee loading provided classification performances of 0.70 (AUC, *p* < 0.05) in distinguishing between knees that developed severe OA or mild OA and knees that did not develop OA eight years after a healthy radiographic baseline. However, FE models incorporating subject-specific femoral and tibial cartilage thickness did not improve this classification performance, suggesting there exists an optimal point between personalized loading and geometry for discrimination purposes. In summary, we proposed a modeling framework that streamlines the rapid generation of individualized knee models achieving promising classification performance while avoiding motion capture and cartilage image segmentation.

**Supplementary Information:**

The online version contains supplementary material available at 10.1007/s10439-024-03549-2.

## Introduction

Osteoarthritis (OA) impairs the correct functioning of synovial joints in the human body, especially the knee, affecting the well-being of millions of people worldwide and causing a high economic burden on societies [29]. Due to its multifactorial nature, developing accurate clinical tools for the prediction of OA is challenging [3]. Nevertheless, computational models have served to study risk factors that contribute to the onset and progression of this disease [38], e.g., joint malalignment [45] and chronic overloading [2] and its treatments [37]. In a previous study [35], we introduced a template-based approach to rapidly create knee finite element (FE) models, simulate their biomechanics, and predict the development of OA over the medial compartment. However, this method has limitations that need to be addressed. For example, it has been validated with a small sample (*N* = 21). It also assumed an even distribution of joint contact forces between the lateral and medial compartments, which is not fully realistic and personalized [45]. In addition, this modeling framework assumes that the fraction of femoral to tibial cartilage thickness in the joint space is constant from the template to the new models.

These limitations open the discussion about the impacts of personalized joint loading and geometry over the predictive capabilities of physics-based methods in cohort studies of OA. In an FE study of two subjects, Wesseling et al., [53] showed that both the loading and geometry affect the acetabular contact pressure in the hip joint, with geometry having a larger influence. In a musculoskeletal and joint contact study of 37 models created from 14 subjects using principal component analysis, Clouthier et al., [9] showed that variations in the local knee geometry may affect the overall functioning of the joint, increasing the risk of developing knee OA for some phenotypes. Similarly, through sensitivity analyses in an FE and experimental study of three knees, Yao et al., [56] concluded that geometry prevails over material formulation on menisci biomechanics, highlighting the use of specimen-specific joint geometries for clinical studies.

Differently from the studies above, other works used their in silico results to predict the future condition of the joints. For instance, in a computational and experimental study of 15 subjects, Aitken et al. [2], calibrated a discrete element analysis-based method to simulate the hip biomechanics and correctly identify the region in the hip prone to degeneration. They used semi-automatically segmented geometries with a representative loading of hip-dysplastic subjects. Finally, in a computational study by discrete element analyses of 38 subjects, Segal et al., [50] indicated that simulated elevated contact stress may serve for predicting the deterioration of cartilage and bone marrow lesions in the knee. They used manually segmented knee geometries and vertically compressed the knees at a flexed angle with half of the body weight of the subject. However, incorporating detailed subject-specific kinematics, kinetics, and joint geometries for numerous subjects is still challenging [38], even more so if the study aims to provide reproducible methods [17]. In this scenario, we believe that approximating individualized knee geometries with simple methods and predicting individualized loading conditions with machine learning tools can overcome these challenges while providing meaningful results to investigate knee OA. To the best of our knowledge, these analyses have not been carried out before.

Regarding geometry, Mononen et al. 2019 compared the OA predictions of models generated from 21 knee geometries obtained via manual segmentation and by scaling a template model based on knee anatomic measurements. They found that by scaling a single template knee geometry, they could predict the future radiographic condition of the knee better than using a fully subject-specific manually segmented knee geometry. The area under the curve (AUC) values comparing osteoarthritis (OA) groups by Kellgren and Lawrence (KL) grades demonstrated this. Using manually segmented geometries models, the AUCs were 0.91 for KL0 vs. KL3, 0.83 for KL0 vs. KL2, and 0.67 for KL2 vs. KL3. However, when employing the template-based method, the AUCs improved substantially, reaching 1.0 for KL0 vs. KL3, 0.89 for KL0 vs. KL2, and 0.88 for KL2 vs. KL3. However, they used the joint space width to scale the femoral and tibial cartilage thickness of the template instead of measuring the thickness separately for both femoral and tibial cartilage. This assumption raises the question about the impact of considering this femoral-to-tibial cartilage thickness ratio on the predictions since cartilage thickness has a significant effect on articular cartilage stresses [1, 41, 48].

Regarding joint loading, the time needed to specify personalized knee joint reaction forces is the main limitation. The golden standard to obtain these forces involves motion capture data followed by musculoskeletal modeling. To overcome this difficulty, multiple machine learning approaches have estimated the kinematics and kinetics of the joints in the lower limbs during walking [6, 15]. Some of them rely on motion data for predicting knee joint contact forces (JCFs), which may still be a limitation for accessibility. In recent studies [26, 27], we developed shallow neural networks for the estimation of the maximum knee JCF during the stance phase of gait. This approach [27] uses simple demographic and biomechanical information of the subjects to predict the maximum total knee JCF (with Pearson correlation between predictions and musculoskeletal model simulations of *r* = 0.8) and corresponding peak contact forces for the medial (*r* = 0.6) and lateral (*r* = 0.7) compartments, avoiding motion capture.

In this work, we aim to further develop and validate a template-based FE approach to predict the development of knee OA. We hypothesize that individualized knee FE simulations through a template-based modeling approach with neural network-based knee JCFs and personalized femoral and tibial cartilage thickness improve the classification of the future radiographic condition of the knees. To this end, we combined novel methods that overcome the previously mentioned limitations of the template-based method with individualized predictions of peak JCFs.

## Materials and Methods

**Fig. 1:**
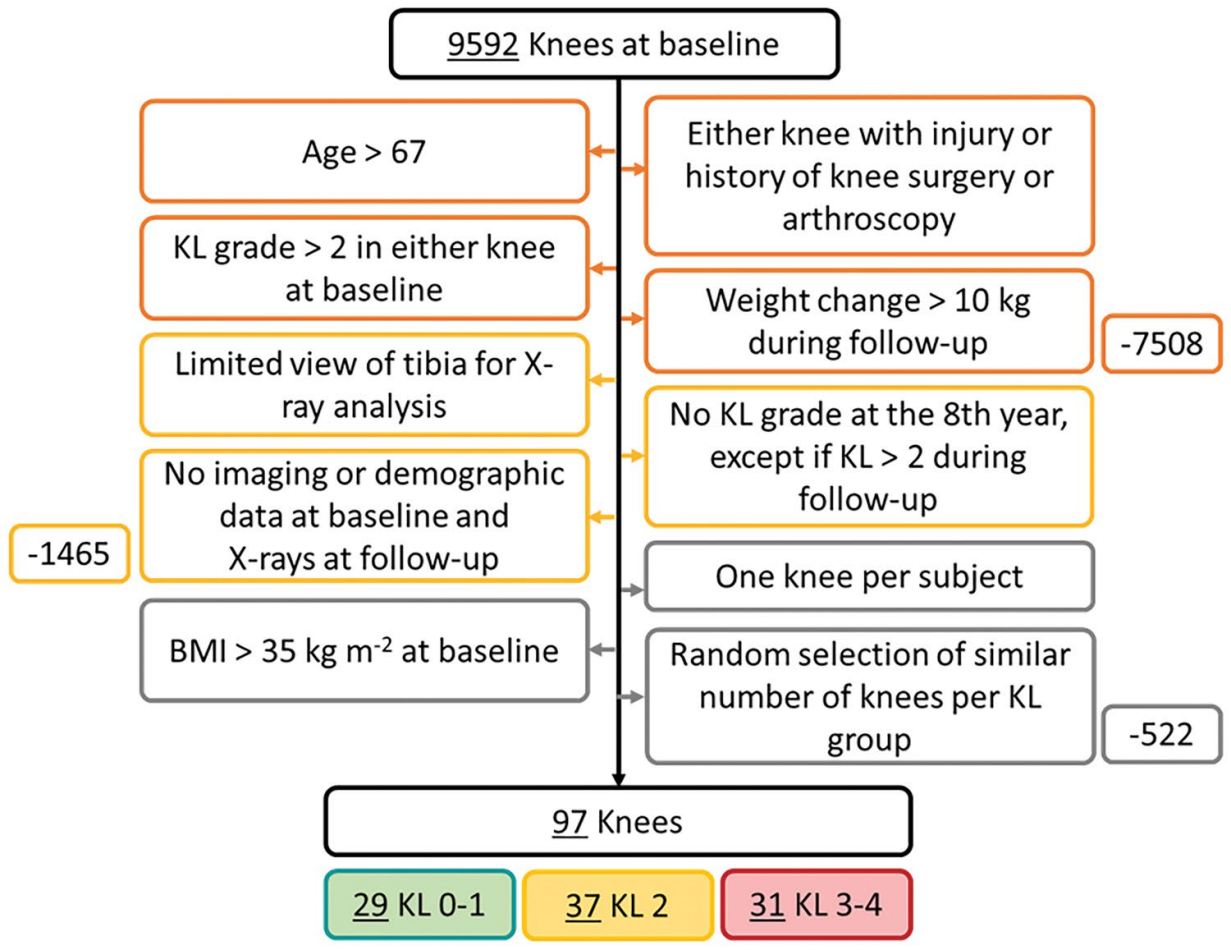
Chart flow describing the procedure to select the knees. Exclusion criteria are in yellow, orange, and gray boxes

We obtained the radiographic and demographic information of 97 knees from the Osteoarthritis Initiative database (OAI, https://nda.nih.gov/oai/), selected according to the exclusion criteria depicted in Fig. 1, at a healthy baseline and after eight years of follow-up. We grouped the knees according to the Kellgren–Lawrence (KL) grade at the follow-up time. We then created medial and lateral compartment models of the knees by scaling a template FE model using information from the healthy baseline and simulated the stance phase of the gait [35]. At this stage, we varied how we defined (i) the maximum tibiofemoral JCF, (ii) the cartilage thickness scaling, and (iii) the thresholds of maximum principal stress for cartilage degeneration.

Next, we compared the FE model outcomes and the classification performance from the different variations in the pipeline. Finally, we correlated our simulated results and the joint space narrowing (JSN) measured between the healthy baseline and the eighth year of follow-up.

### Subject Characteristics

Table 1 presents the baseline characteristics of the subjects and their knees. Out of the 97 subjects, 71% were female, and no differences were observed among the variables for the different KL groups.

**Table 1:** Baseline demographic and anatomic measurements of the subjects and their knees, respectively

Variable	KL group				*p* value
	KL 0–1 *n* = 29	KL 2 *n* = 37	KL 3–4 *n* = 31	All *n* = 97	
Right knees	17 (58%)	21 (57%)	17 (55%)	55 (57%)	–
Sex, female	18 (62%)	29 (78%)	22 (71%)	69 (71%)	–
Age (years)	58.00 [50.75–62.00]	57.00 [53.75–58.25]	60.00 [56.25–62.75]	58.00 [53.75–61.25]	0.122
Mass (kg)	72.90 [63.17–86.13]	76.40 [63.05–87.20]	73.60 [67.47–90.00]	73.60 [64.35–88.32]	0.561
Height (cm)	168.4 [163.5–173.6]	163.4 [158.5–172.7]	166.3 [159.5–171.9]	166.5 [159.9–172.9]	0.252
Walking speed (m s^−1^)^a^	1.38 [1.27–1.55]	1.31 [1.25–1.43]	1.40 [1.30–1.53]	1.37 [1.25–1.50]	0.124
Joint alignment (deg)^b^	6.18 [3.35–7.61]	4.47 [2.95–6.37]	5.22 [1.86–7.56]	5.14 [2.49–7.21]	0.333
ICD (mm)	40.60 [38.50–45.32]	39.20 [37.62–42.17]	40.60 [38.50–42.70]	40.60 [37.8–43.4]	0.189
Medial A-P (mm)	54.36 [52.06–57.03]	52.63 [51.05–56.86]	52.88 [50.20–54.56]	53.05 [51.05–56.42]	0.213
Lateral A-P (mm)	63.53 [61.71–67.81]	61.38 [59.14–67.41]	61.62 [59.38–64.35]	62.59 [59.62–65.88]	0.051
Medial JS (mm)	4.74 [4.40–5.44]	4.98 [4.36–5.54]	4.97 [4.37–5.47]	4.79 [4.37–5.48]	0.711
Medial femoral thickness (%)^c^	57.00 [53.75–60.00]	53.00 [47.00–57.00]	54.00 [48.50–57.00]	55.00 [48.00–57.25]	0.095
Lateral JS (mm)	5.38 [5.02–6.15]	5.27 [4.83–6.20]	5.75 [4.65–6.31]	5.38 [4.74–6.20]	0.978
Lateral femoral thickness (%)	42.00 [37.75–46.25]	40.00 [34.00–45.25]	42.00 [37.25–49.25]	40.00 [36.00–46.00]	0.411

Values show median and interquartile range [25–75%]

*ICD* intercondylar distance, *A-P* anterior–posterior distance, *JS* joint space. *p* value from Kruskal–Wallis statistical test comparing KL groups

^a^Subject self-selected walking speed reported in the OAI for a 20 m distance

^b^X-rays of frontal plane joint alignment reported in the OAI

^c^Percentage of joint space corresponding to the femoral cartilage

### Finite Element Models

**Fig. 2:**
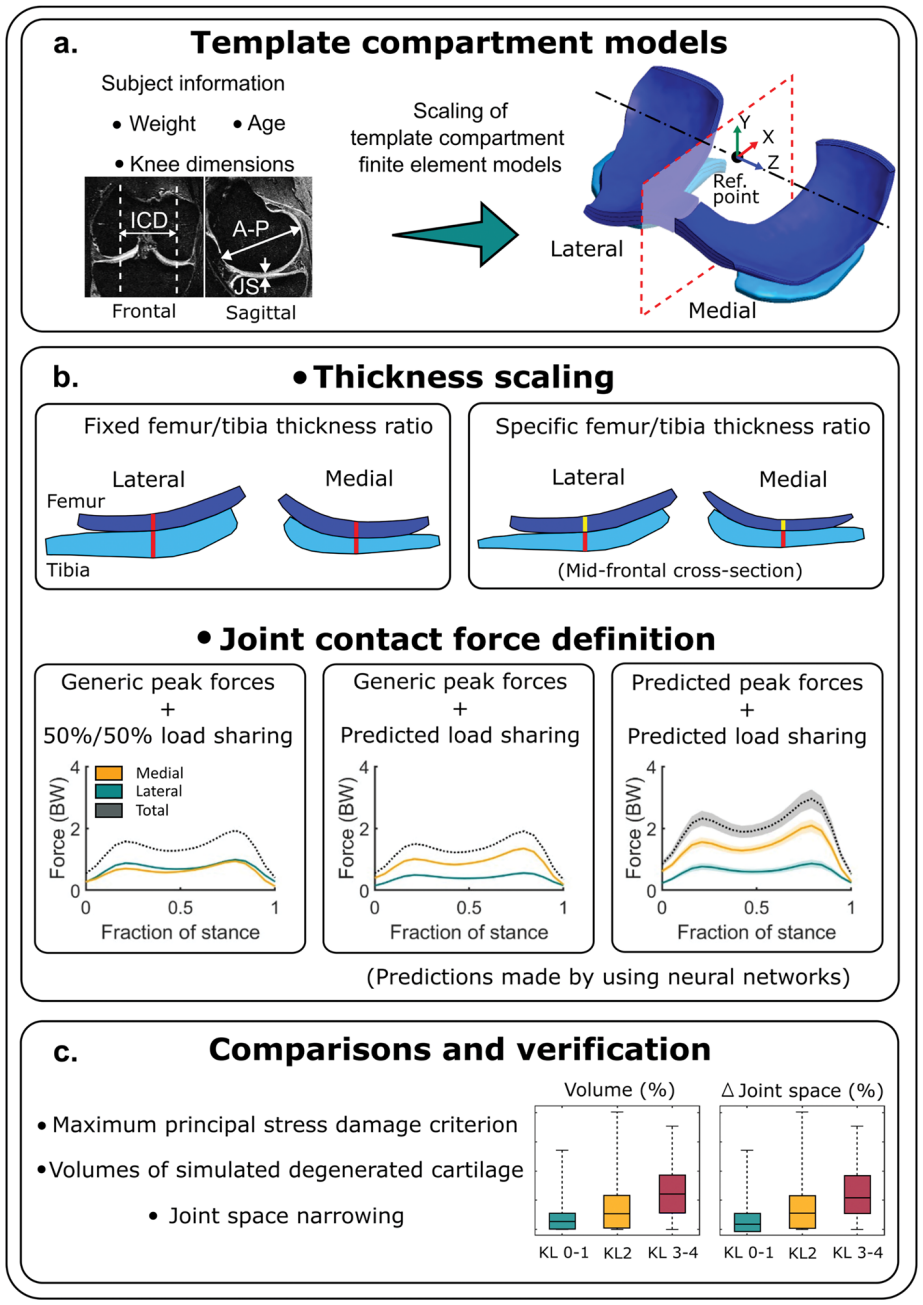
Workflow of the present study. **a** Generation of knee joint compartment models using simple MRI-based measurements. **b** Parameters varied in the template-based method: thickness scaling (top) and loading definition (bottom). **c** Comparison of the predicted degenerated volumes of cartilage tissue and verification against experimental observations. *ICD* intercondylar distance, *A-P* anterior–posterior distance, *JS* joint space

We implemented a template-based modeling method [36] for the lateral compartment as described previously for the medial compartment (Fig. 2a). Here, to generate individualized compartment models, template FE models of the lateral and medial compartments (single template per compartment), incorporating their corresponding geometries and loading conditions, were scaled based on the information obtained from the subject of interest. The template geometry comprised the femoral and tibial cartilages and considered the other tissues in the knee joint through boundary conditions [35, 36].

#### Geometry and Loading

In brief, to generate the geometry, we scaled a single template FE mesh using the ratio between simple MRI-based anatomic measurements from the template knee and the knee to be modeled. After a mesh convergence analysis, the template FE mesh was composed of 18471 linear hexahedral elements in the medial compartment and 17010 elements in the lateral compartment The average edge length of the elements was 0.6 mm, and the aspect ratios were (median [interquartile range]) 1.81 [1.62–2.03] for the medial compartment and 2.04 [1.78–2.37], for the lateral compartment. The anatomic measurements included the femoral intercondylar distance (ICD), maximum femoral anterior–posterior (A-P) distances from the medial and lateral condyles, and the joint space (JS) measured in the same planes as the A-P distances. The A-P and ICD measurements were used to scale the geometry in the X- and Y-directions, respectively (Fig. 2a). The JS measurements were used to scale the tibial cartilage thickness in the Y-direction and radially for the femoral cartilage [36]. Loading was applied to the models by pairing the knee JCF in the axial direction (Y-axis) with the flexion angle (Z-axis) during the stance phase of gait [35]. In this work, to individualize the knee loading, an experimentally determined shape of JCF [24] was scaled to match peak JCFs predicted by three different methods, while the flexion angle trajectory was kept fixed. The *Variations overview* section describes these methods.

#### Cartilage Model

The cartilage model followed a biphasic and fibril-reinforced formulation (Table 2) implemented in FEBio 3.7.0 [31] with a depth-dependent arcade-like orientation of collagen fibrils [4, 42]. This formulation allowed us to implement tensile stress thresholds to define the damage onset in the cartilage collagen network. When compressed, this biphasic and fibril-reinforced formulation pressurizes the fluid phase and stretches the stiff collagen fibril network, contributing strongly to the dynamic stiffness of cartilage [12]. The contact between cartilages was defined as *sliding elastic* [32], which constrains the fluid exudation from the tissue, assuming negligible fluid exchange during the simulated time of approximately one second [13].

**Table 2:** Cartilage model material properties

Property	Tibia	Femur	Model representation
${\xi }_{\text{fp}}$(MPa)^a^	32	215	
${\beta }_{\text{fp}}$(−)^a^	2.6	2.6	
${\xi }_{\text{fs}}$(MPa)^a^	1.0	3.0	
${\beta }_{\text{fs}}$(−)^a^	2.6	2.6	
${E}_{\text{nf}}$(MPa)^b^	0.106	0.215	
${\nu }_{\text{nf}}$(−)^b^	0.15	0.15	
${\kappa }_{0}$(10^−15^ m^4^ N^−1^ s^−1^)^b^	18	6	
M (−)^b^	15.24	5.06	
${\varphi }_{0}$(−)^c^	0.2	0.2	

^a^Paz et al., 2022, ^b^Julkunen et al. 2007, ^c^Holmes and Mow 1990. ${\xi }_{\text{fp}}$ stiffness parameter of primary fibrils, ${\xi }_{\text{fs}}$, stiffness parameter of secondary fibrils, ${\beta }_{\text{fp}}$ is the power of primary fibrils, ${\beta }_{\text{fs}}$ is the power of secondary fibrils, ${\text{E}}_{\text{nf}}$ is Young’s modulus of the isotropic ground matrix, and ${\text{v}}_{\text{nf}}$ its Poisson’s ratio, ${\kappa }_{0}$ initial hydraulic permeability, M, the material parameter for the strain-dependent model of permeability, and ${{\varphi }}_{0}$, initial solid fraction.

### Variations Overview

We combined two different approaches to scaling the cartilage thickness and three ways to define the loading of the knee compartments in the template-based method (Fig. 2b; Table 3). In addition, we examined two different equations defining thresholds of maximum principal stress for the initiation of cartilage degeneration.

**Table 3:** Abbreviations used for cartilage thickness scaling and compartmental joint contact force definitions

	Abbreviation	Description
Cartilage thickness scaling method	Fixed thickness ratio	Keeps constant the femoral-to-tibial cartilage thickness ratio of the template model
	Scaled thickness ratio	Uses the femoral-to-tibial cartilage thickness ratio of the modeled knee
Joint contact force definition	50%/50%	Asses an even sharing of JCF between compartments (50% for the medial and 50% for the lateral compartment), with maximum JCFs obtained by scaling an experimentally determined gait load curve (Kutzner et al. 2017) by the body weight of the subjects
	LS—NN	Uses a load sharing (LS) between the medial and lateral compartment predicted by neural networks (NN), with maximum JCFs obtained by scaling an experimentally determined gait load curve using the body weight of the subjects
	LS & Peak—NN	Uses predicted load sharing (LS) and peaks of tibiofemoral JCF by artificial neural networks (NN)

We used two methods to scale the template cartilage thickness using MRI measurements, as shown in Table 3. By this, we aimed to determine whether using the joint space, keeping the femoral-to-tibial cartilage thickness ratio constant, provides similar predictive results compared to personalizing the femoral-to-tibial cartilage thickness ratio by measuring the femoral and tibial cartilage thickness separately. The former could be possible by X-rays [21], which are more affordable and faster than MRI but unable to directly show cartilage thickness for the femur and tibia separately, and the latter only by MRI.

Regarding the three different approaches we used to define the peak JCFs (Table 3), we wanted to investigate whether using neural network-based peak JCFs and load share between knee compartments would improve the OA predictions compared to simpler assumptions [35, 36]. In the first two cases, we scaled an experimentally determined peak JCF to the subjects using their body weight. In one case, we evenly divided the peak JCF between knee compartments (50%/50%). In the other case, we implemented a load sharing predicted by neural networks (LS—NN). Finally, in the third case, we used load sharing and peak JCFs predicted by neural networks (LS & Peak—NN). See the *Neural network predictions* section for further details.

With respect to the tissue degeneration mechanism, we assumed that the deterioration in the cartilage tissue begins once the collagen fibrils exceed age-dependent thresholds of tensile stress. We implemented two approaches to evaluate the sensitivity of the response variable to these thresholds. See the *Degeneration model* section.

### Neural Network Predictions

We used feed-forward artificial neural networks (NN) to individualize the peak compartmental knee JCFs [26, 27]. The predictors comprised the height, weight, walking speed, joint frontal alignment, age, and sex of the subjects, and the outputs consisted of the medial (*r* = 0.61 ± 0.15), lateral (*r* = 0.67 ± 0.13), and total (*r* = 0.80 ± 0.10) maximum JCFs during the stance phase of the gait. The load sharing was computed as the ratio of the medial and lateral maximum JCFs to the total predicted maximum knee JCF.

### Degeneration Model

First, we defined the stress thresholds for degeneration (${T}_{{\sigma }_{\text{f}}}$) based on experimental observations by Kempson on monotonic tensile tests of human cartilage samples [23, 35]

1 $$T_{{\sigma_{{\text{f}}} }} = \left\{ \begin{gathered} \begin{array}{*{20}c} {30\;{\text{MPa}},} & {{\text{Age}} \le 30} \\ \end{array} \hfill \\ \begin{array}{*{20}c} {\left( {30 - \left( {{\text{Age}} - 30} \right)\left( {20/15} \right){ }} \right)\;{\text{MPa}},} & {30 < {\text{Age}} \le 45} \\ \end{array} \hfill \\ \begin{array}{*{20}c} {\left( {10 - \left( {{\text{Age}} - 45} \right)\left( {3/20} \right){ }} \right)\;{\text{MPa}},} & {45 < {\text{Age}} \le 65} \\ \end{array} \hfill \\ \begin{array}{*{20}c} {\left( {7 - \left( {{\text{Age}} - 65} \right)\left( {2/100} \right){ }} \right)\;{\text{MPa}},} & {65 < {\text{Age}} \le 75} \\ \end{array} \hfill \\ \begin{array}{*{20}c} {6.8\;{\text{MPa}},} & {{\text{Age}} > 75} \\ \end{array} \hfill \\ \end{gathered} \right..$$

Second, we used the relationship proposed by Weightman et al. 1978, between the age of the donors, the cyclic tensile stress in human cartilage samples (${T}_{{\sigma }_{\text{f}}}$), and the number of cycles to failure ($N$)

2 $$T_{{\sigma_{{\text{f}}} }} = \left( {25.4{ } - 0.15\;{\text{Age }} - 1.65\log_{10} \left( N \right){ }} \right)\;{\text{MPa}}$$

The latter, with three representative numbers of cycles: *N* = 10^5^ accounting for low cycle fatigue and *N* = 10^6^ and *N* = 10^7^ representing 5000–50000 steps a week for eight years [47]. For simplicity, in the *Results *section, we show outcomes from the models using Eq. 2 with *N* = 10^6^, and the others are presented in the *Supplementary material*.

### Quantification of Degenerated Tissue

**Fig. 3:**
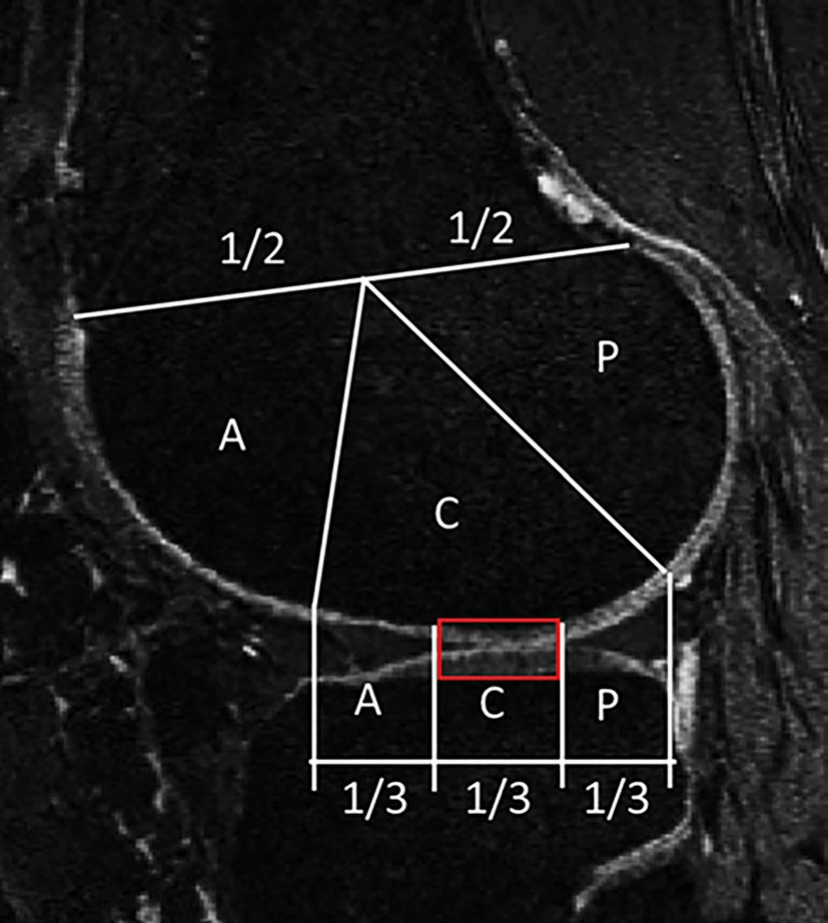
MRI sagittal showing in red the region of interest used to define the volumetric measure of cartilage degeneration. *A* anterior, *C* central, *P* posterior

To quantify the simulated degeneration, we summed up the volume of the elements exceeding the age-dependent thresholds for degeneration during the stance phase of gait [35, 36, 41] and divided it by the reference volume shown in Fig. 3. We limited our analysis to the central portion of the joint, following the MOAKS joint partitioning [19], as the FE models were constrained to simulate the contact between cartilages in this region.

### Verifications

We not only evaluated how well the template method predicts future KL grades but also looked at how closely the simulated results correlate with other ways of measuring the gradual changes in knee OA. To this end, we compared the predicted volumes of degenerated tissue against the joint space narrowing (JSN), assuming the JSN is an indirect measurement of alterations in knee structures [16, 20].

We defined the JSN as the percentage difference in joint space width (JSW) between the healthy baseline and after 8 years of follow-up, measured in the medial and lateral compartments from load-bearing frontal plane X-rays. We defined the JSW as the vertical distance between the central point of the surface of femoral condyles projected to the tibial plateaus [43]. We implemented an in-house MATLAB (The MathWorks, Natick, MA, USA) tool for the semi-automatic measurement of JSW.

### Statistical Analysis

We opted for non-parametric analyses because the data were not normally distributed and did not have homogeneous variance. We compared the simulated degeneration between the different combinations of loading and thickness scaling by Friedman tests, between the medial and lateral compartments by Wilcoxon signed rank test and between KL groups at the eighth year of follow-up by Kruskal–Wallis tests. In cases with multiple comparisons, we applied the Bonferroni correction to adjust the level of significance *α* = 0.05. To assess how the different combinations of loading, geometry, and thresholds for degeneration influenced the predictive capabilities of the method, we used the area under the curve (AUC) of logistic receiver operating characteristic (ROC) analyses, using the simulated degenerated volumes at the baseline time. This metric is easy to interpret [33], and it has been used before in knee OA predictions to compare the performance across classification algorithms [44]. We used DeLong’s criterion to evaluate the significance of AUCs [10]. We used adjusted-R^2^ measure and cross-tabulation analysis to evaluate if the compartment with the largest simulated degeneration in the knee correlates with the compartment with the largest JSN in corresponding knees. DeLong’s criterion was implemented in R (v.4.3.1, http://www.r-project.org/) [40] and the other statistical analyses in MATLAB 2022b.

## Results

**Fig. 4:**
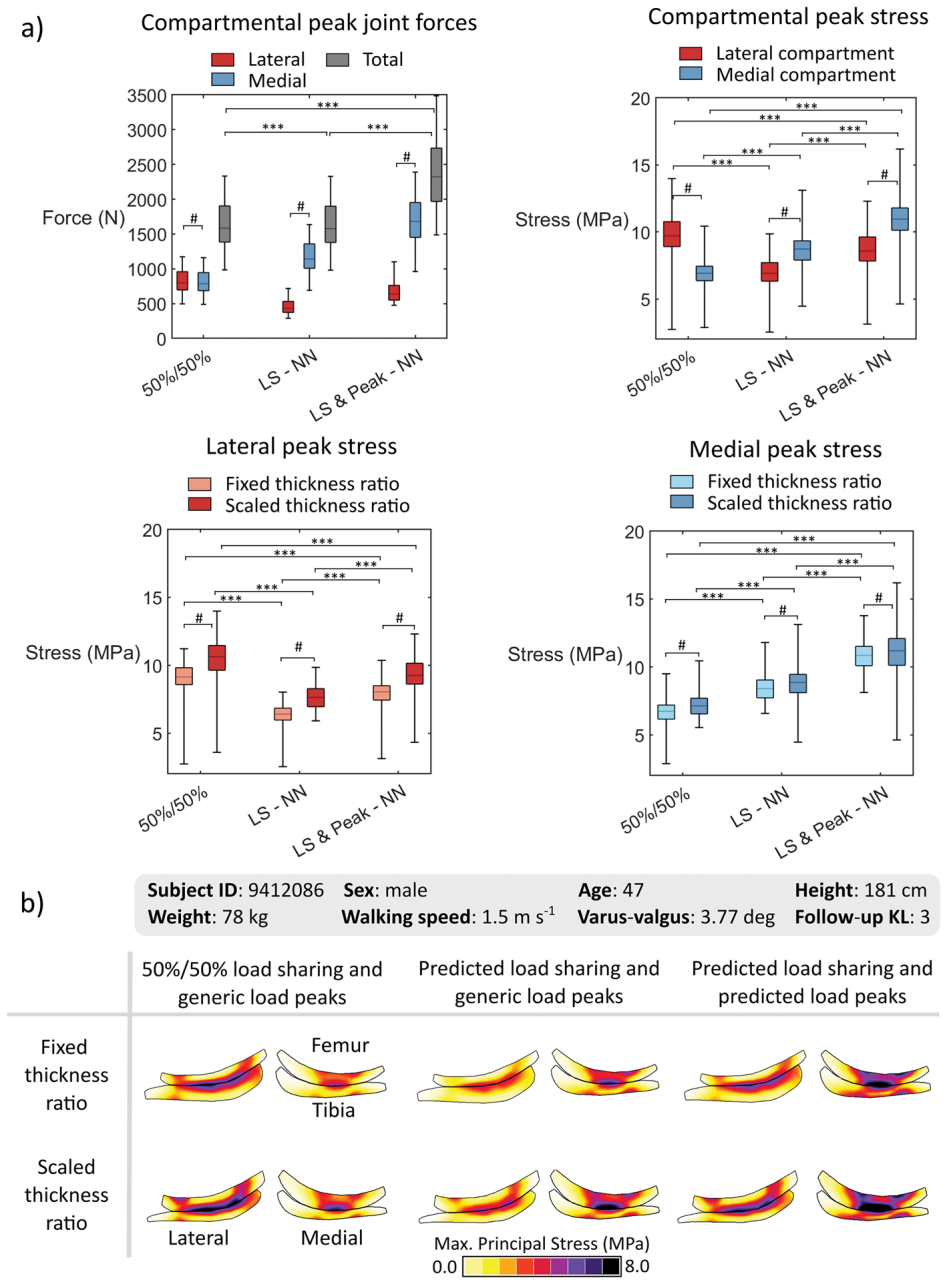
**a** Peak joint contact forces (top, left) and maximum principal stresses in the tibial cartilage for the medial and lateral compartments during the first half of the stance (top, right), and the lateral (bottom, left) and medial (bottom, right) compartments for the models with the fixed and scaled cartilage thickness ratios. **b** Distribution of maximum principal solid stress in the mid-frontal cross-section of one knee model at the loading response of the stance. The columns represent loading conditions, and the rows represent thickness scaling methods. 50%/50%—generic evenly distributed joint contact force, LS-NN—load sharing predicted by neural networks with maximums from a generic curve, LS & Peak-NN—load sharing and peak forces predicted using neural networks. ***p < 0.001, #*p* < 0.05.

**Fig. 5:**
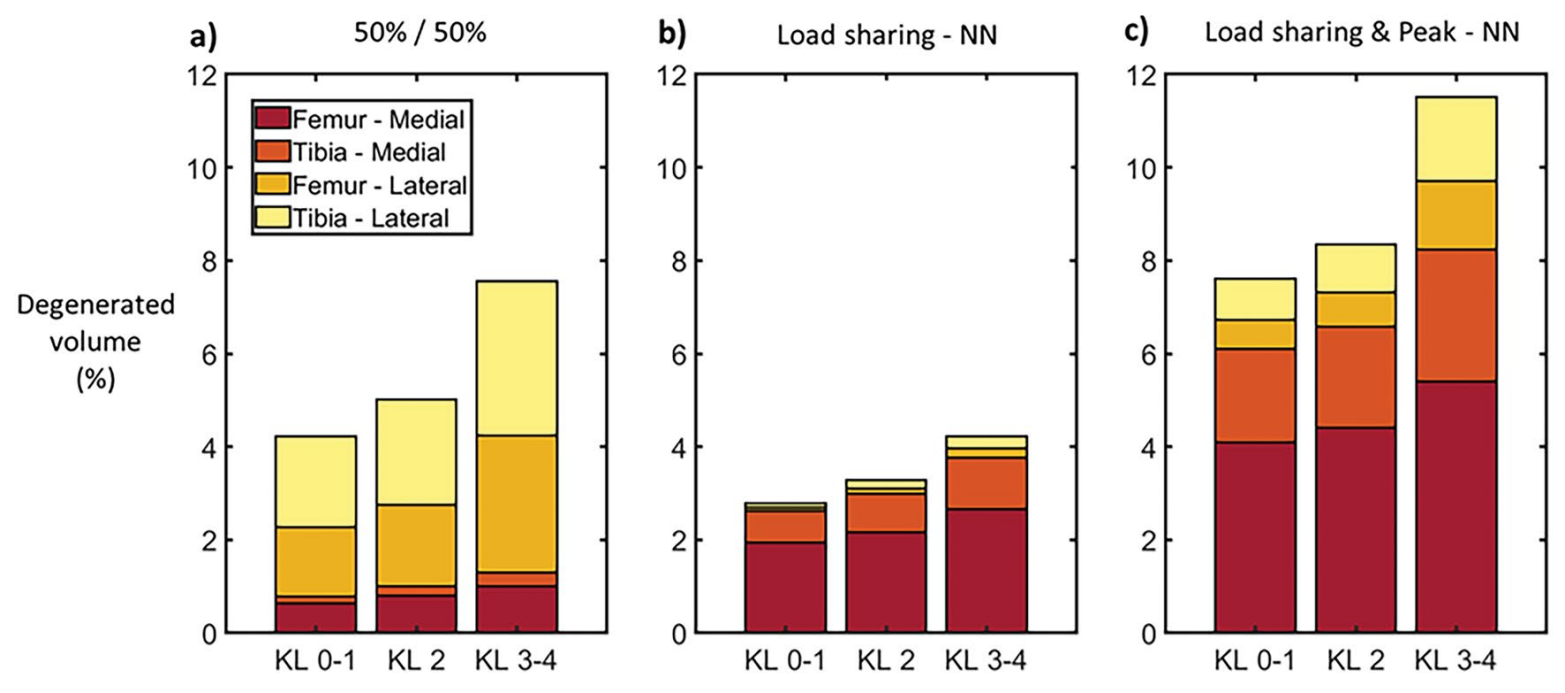
Average contribution of medial and lateral compartments of tibial and femoral cartilages to the simulated overall degenerated volume using **a** a 50%/ 50% loading distribution between compartments and peak joint contact forces from the literature, **b** loading distribution estimated by neural networks (NN) and peak joint contact forces from the literature, and **c** load sharing and peak joint contact forces estimated by neural networks. Volumes correspond to age-dependent damage thresholds given by Eq. 2 with *N* = 10^6^ cycles on models using the femoral-to-tibial cartilage thickness ratio of the template model.

Figure 4 shows that all the varied parameters modified the stress distributions. However, the variations in loading caused a larger impact on tensile stress distributions compared to variations in the thickness scaling method. Using an evenly distributed JCF between compartments caused higher stresses in the lateral compartment. Using predictions of peak JCFs by neural networks increased overall stresses in the knee and shifted the highest values to the medial compartment, compared to using an even distribution of load sharing and peak JCF from the literature. Figure 5 shows the corresponding degenerations caused by the variations in loading in each cartilage and compartment.

**Fig. 6:**
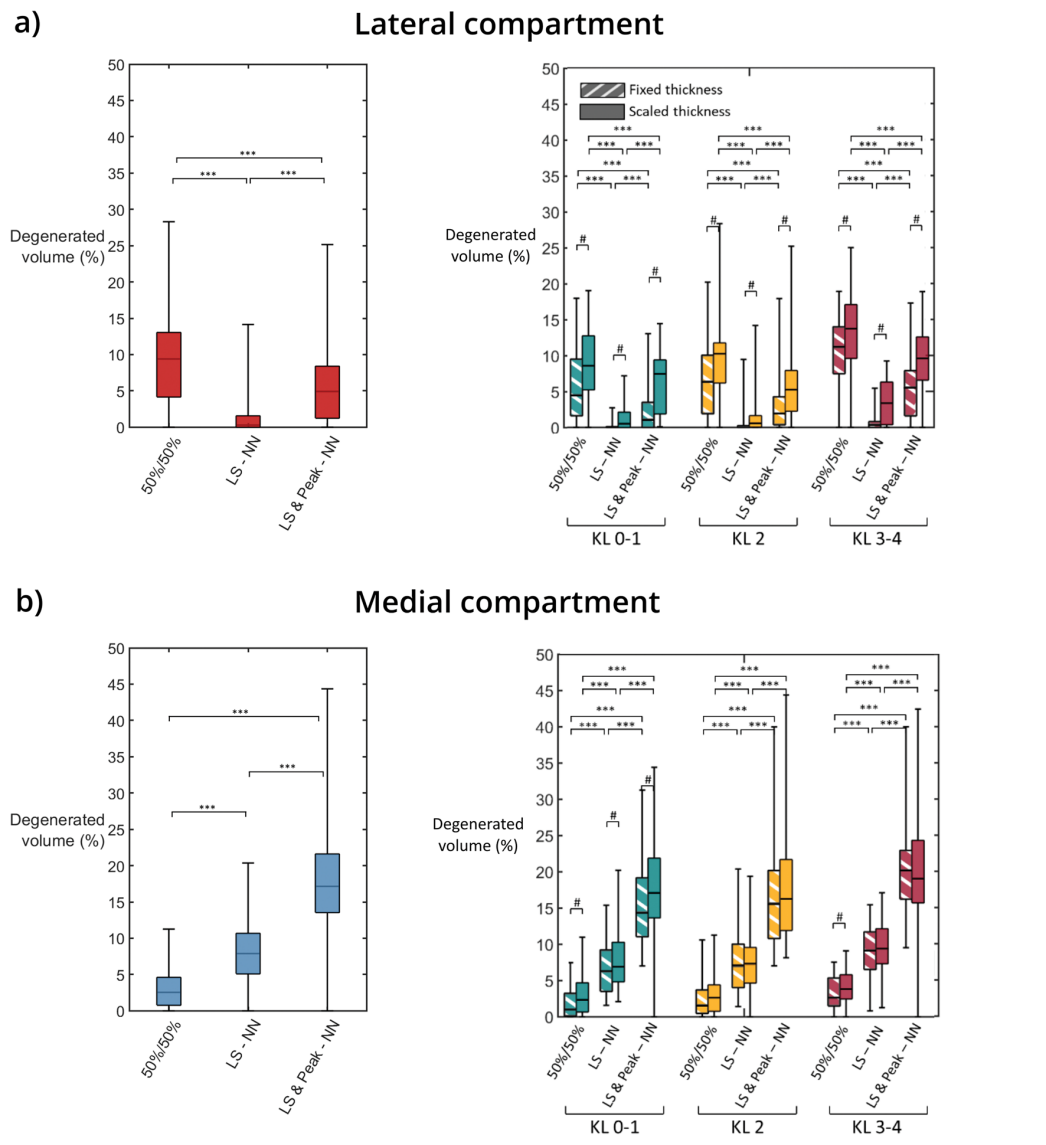
Simulated degenerated volume of cartilage for the **a** lateral and **b** medial compartments, using Eq. 2 with *N* = 10^6^. On the left, volumes pooled by force definition, and on the right, volumes pooled by thickness scaling method and Kellgren–Lawrence (KL) grade. Boxes show the first, second, and third quartiles, and whiskers represent the range. 50%/50%—generic evenly distributed joint contact force, LS-NN—load sharing predicted by neural networks with maximums from a generic curve, LS & Peak-NN—load sharing and peak forces predicted using neural networks. ***p < 0.001, #*p* < 0.05.

Regarding simulated cartilage degeneration for the lateral compartment (Fig. 6a), the generic model with an even distribution of JCF between compartments and the maximum JCF from the literature (50%/50%) yielded the largest degenerated volumes compared to the other models (*p* < 0.001). In contrast, in the medial compartment (Fig.6b), using peak JCFs from the literature resulted in lower degenerated volumes compared to the model with predicted peak JCFs by neural networks (*p* < 0.001).

**Fig. 7:**
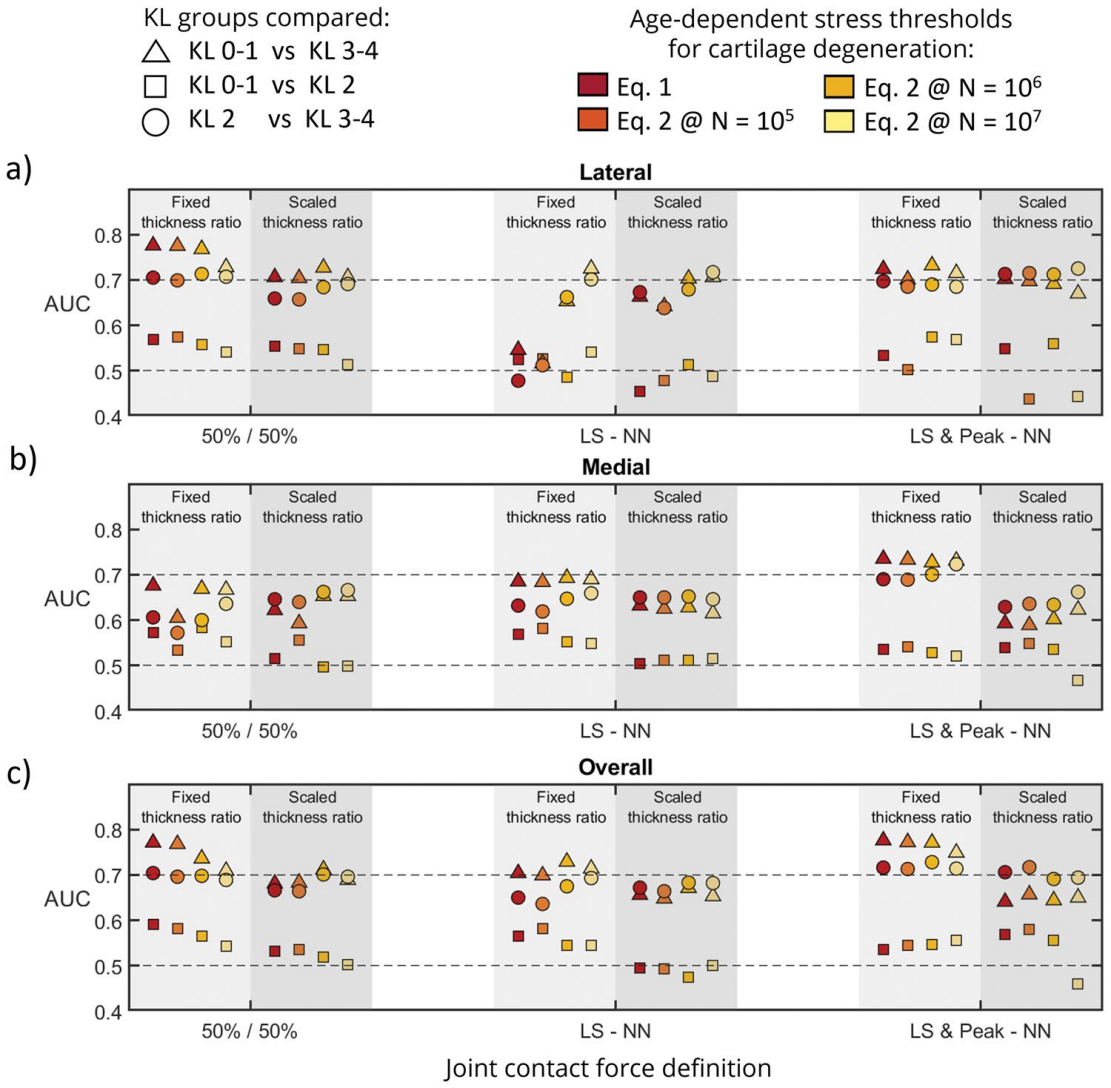
Areas under the curve (AUCs) from comparing the different pairs of KL grades (KL 0–1 vs KL 2 in squares, KL 0–1 vs KL 3–4 in triangles, and KL 2 vs KL 3–4 in circles) for each of the models, considering the degenerated volumes from **a** the lateral compartment, **b** the medial compartment, and **c** the overall knee. Each color represents an age-dependent stress threshold function for degeneration. Dashed lines represent reference AUCs of 0.5 and 0.7 for the stratification by the models to different KL grade groups. Additional comparisons can be found in the *Supplementary material*.

Based on the AUC analysis, our method was unable to differentiate between KL 0–1 and KL 2 groups for any of the parameter combinations (Table 4). Overall, implementing personalized thickness scaling for femoral and tibial cartilages did not improve the AUCs (Fig. 7) compared to using the JS and the femoral-to-tibial cartilage fraction from the template. Overall, the model with NN-based compartmental JCFs and the thickness ratio from the template best discriminated the knees using either volume from the lateral, medial, or overall knee (median AUC = 0.70, range [0.50–0.78]), for all the implemented failure thresholds (Eqs. 1 and 2 with *N* = 10^5^, 10^6^, and 10^7^). See the *Supplementary material* for further comparisons.

**Table 4:** Areas under the curve^a^ of logistic ROC analysis, in parenthesis the *p* values by the DeLong criterion

Compartment	Lateral			Medial			Overall		
Model	KL0–1 vs KL2	KL0–1 vs KL3–4	KL2 vs KL3–4	KL0–1 vs KL2	KL0–1 vs KL3–4	KL2 vs KL3–4	KL0–1 vs KL2	KL0–1 vs KL3–4	KL2 vs KL3–4
50%/50% Fixed thickness ratio	0.557	0.768	0.713	0.583	0.669	0.600	0.565	0.736	0.698
	(0.427)	**(0.000)**	**(0.001)**	(0.250)	**(0.018)**	(0.152)	(0.374)	**(0.000)**	**(0.002)**
50%/50% Scaled thickness ratio	0.547	0.727	0.684	0.497	0.653	0.662	0.519	0.712	0.701
	(0.520)	**(0.001)**	**(0.007)**	(0.964)	**(0.035)**	**(0.015)**	(0.799)	**(0.002)**	**(0.002)**
LS—NN Fixed thickness ratio	0.485	0.653	0.662	0.552	0.693	0.647	0.545	0.729	0.675
	(0.809)	**(0.026)**	**(0.011)**	(0.477)	**(0.006)**	**(0.030)**	(0.535)	**(0.001)**	**(0.008)**
LS—NN Scaled thickness ratio	0.513	0.703	0.679	0.511	0.628	0.652	0.474	0.671	0.683
	(0.865)	**(0.003)**	**(0.009)**	(0.885)	(0.080)	**(0.024)**	(0.729)	**(0.016)**	**(0.006)**
LS & Peaks—NN Fixed thickness ratio	0.575	0.732	0.690	0.528	0.727	0.700	0.547	0.771	0.728
	(0.304)	**(0.000)**	**(0.005)**	(0.696)	**(0.001)**	**(0.002)**	(0.514)	**(0.000)**	**(0.000)**
LS & Peaks—NN Scaled thickness ratio	0.560	0.691	0.712	0.535	0.602	0.634	0.555	0.644	0.691
	(0.417)	**(0.005)**	**(0.001)**	(0.635)	(0.160)	(0.052)	(0.460)	**(0.044)**	**(0.003)**

Analysis was done using the failure line described by Eq. 2 associated with *N* = 10^6^ cycles. The bold font highlights significant AUCs (*p* < 0.05)

^a^An area of 1.0 indicates perfect differentiation between the groups compared and an area of 0.5 indicates a random distribution of volumes between the groups. 50%/50%—even load distribution between medial and lateral compartments, LS-NN—load sharing predicted by neural networks, LS & Peaks-NN—load sharing, and peak JCF predicted by neural networks.

**Fig. 8:**
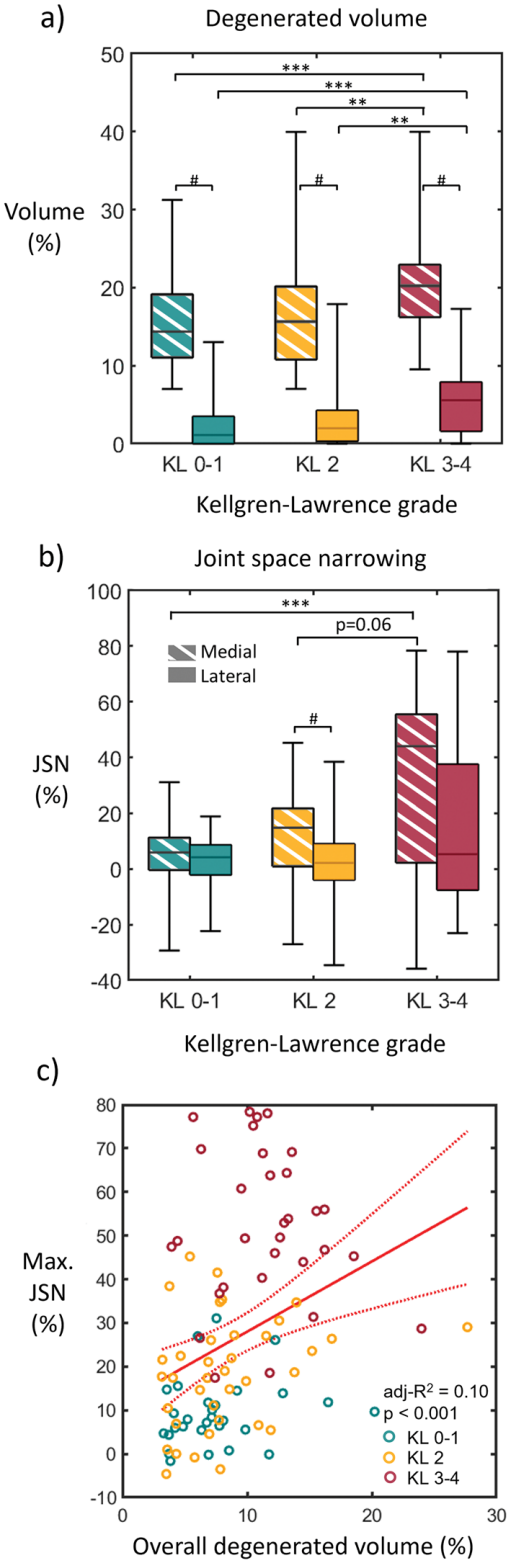
Comparison of the **a** simulated degenerated volumes, **b** joint space narrowing, and **c** linear regression between the maximum joint space narrowing in the knee and the overall joint simulated degenerated volume. These results correspond to using LS & Peaks-NN with scaled thickness ratio and Eq. 2 with *N* = 10^6^ cycles. *p < 0.05, ***p < 0.001, #*p* < 0.05.

The model with NN-based JCF predicted 2.6 [2.0–4.2] (median [interquartile range]) times higher simulated degenerated volumes for the medial compartment compared to those for the lateral compartment (*p* < 0.001) (Fig. 8). In addition, the simulated degeneration in both compartments of severely affected joints (KL 3–4) differed significantly from the healthy (KL 0-1) and mildly (KL 2) affected joints (*p* < 0.001). In contrast, only the JSN (Fig. 8b) in the medial compartment significantly differed between the KL 0–1 and KL 3–4 groups (*p* < 0.001). Regarding compartmental JSN differences, it was higher in the medial compartment only in the mildly affected joints (*p* = 0.025). Linear regression and cross-tabulation analyses suggested that the simulated degenerations do not explain the JSN, with an adj-R2 = 0.104 (*p* < 0.001) and the model constantly predicting larger damaged volumes in the medial than lateral compartment.

## Discussion

In the present study, we extended the template-based method for the simulation of the knee and the prediction of OA. To this end, we simulated 97 knees, modeling the lateral and medial compartments of the tibiofemoral joint, incorporating neural network-based predictions of peak knee JCFs, evaluating two ways of specifying cartilage thickness, and evaluating four age-dependent thresholds for cartilage degeneration. We found that loading should be considered with special care. Assuming equal contributions from the compartments to withstand the knee JCF resulted in persistently elevated stresses in the lateral compartment and subsequent higher lateral degenerations (Figs. 4, 5). In contrast, models using compartmental load sharing by neural networks resulted in simulated degenerations more consistent with the literature, i.e., OA is more frequent in the medial than in the lateral compartment of the knee [54].

AUC analyses showed that including the lateral compartment in the predictions enhanced the classification performance compared to using the results from the medial compartment only (Table 4; Fig. 7). This could be attributed to the anatomic differences between the medial and lateral compartments and the possibility of using more realistic loadings when both compartments are considered. The geometry of the lateral compartment is less congruent compared to the medial compartment [39], causing the stresses in this compartment to be more sensitive to variations in geometry and loading (Figs. 5, 6). This characteristic caused the lateral femoral and tibial cartilages to similarly contribute to the amount of overstressed tissue, differently to the medial compartment where the femoral cartilage has a larger contribution (Fig. 5).

In the verification, we observed that for the medial compartment, simulated degenerated volumes and JSN differed between KL 0–1 and KL 3–4 groups. However, these parameters were weakly correlated (Fig. 8). One possible reason for this is that JSN is associated with changes in other joint structures, besides cartilage tissue, that we are not including in the models, e.g., meniscal extrusions [16, 20, 43]. Then, it would be valuable to validate our predictions against the loss of cartilage volume measured in the respective knee compartments by MRI [8, 55]. In addition, other modeling-based outcomes could be tested aiming to better conjugate the predictive and explanatory capabilities of the method, like cumulative exposure of cartilage [2, 34]. Preliminary tests with this digital biomarker (not shown) provided similar group-wise differences.

In this study, the three methods we considered to individualize the peak JCF experienced by the knee compartments during normal walking had significant effects on the simulated degenerations (Fig. 6). However, it is a challenging task to validate these force magnitudes against experimental measurements in uninjured knees because: firstly, the experimentally obtained generic JCF, used in the 50%/50% and LS–NN loading models, corresponded to data from subjects with instrumented knee arthroplasties and, secondly, the neural networks were designed to predict simulated data from musculoskeletal models. Despite these limitations, these three methods induced cartilage stress peaks similar to those reported in experimental studies [14, 18]. Furthermore, these methods provided means to approximate the personalized loading in the knee using simple demographic and biomechanical data comprising age, weight, gender, height, joint alignment, and walking speed [26, 27, 46].

We identified the challenging need for more recent data characterizing the damage mechanisms of articular cartilage under cyclic loading spanning a wide range of subject ages. While the works by Kempson [23] and Weightman et al. [51, 52] studied the tissue from an engineering perspective, characterizing the tensile mode of failure, we believe that studying mixed loadings (e.g., compressive, sliding, shear, and tensile stresses), including biological aspects of the tissue surroundings, e.g., the concentration of inflammatory factors [22, 28], could provide a more comprehensive understanding of damage mechanics with aging in the cartilage constituents.

In this study, we want to highlight three aspects of using physics-based models. Firstly, in addition to the measurable changes in the outcomes from modeling subjects under different risk factors, the results can be interpreted to predict the future condition of the joint, as demonstrated in the present study and others [2, 49]. This predictive capability can aid in better understanding disease progression mechanisms. Secondly, physics-based models can be intuitively modified to quantify the effect of variations of the different parameters involved in the workflow, e.g., uncertainty in tissue material properties [11]. This allows for sensitivity analyses and clarity of the influence of different factors on the outcomes. Finally, these methods could be further developed to model the effects of preventive and conservative treatments, e.g., weight loss and changes in JCF by gait retraining [30], providing valuable insights into the potential benefits of non-surgical interventions and guiding treatment decision-making.

There are several limitations to our study. For instance, we did not explicitly consider the geometry of the menisci but only their load-bearing function, by reducing the cartilage–cartilage JCF in the corresponding compartments [35]. Our method also currently ignores the varying characteristics of the gait load curve with age, which could potentially impact the amount of tissue at risk [5]. For instance, it is known that, in elders, the second peak of JCF during the stance phase of gait is lower, the midstance JCF is higher, and the walking speed is slower than in young people [7]. These alterations may increase cartilage stress exposure, promoting the damage of tissue constituents. In addition, we did not model all the relationships between the local morphology of the joint and its effects on loading [9]. Another limitation consisted of using the same material properties of articular cartilage for all the knees [25]. Yet, it is still challenging to measure and translate these variations into numerous models.

We showed how physics-based methods can be expedited using estimates of joint loading obtained from machine learning tools, avoiding cumbersome motion capture procedures. We accounted for individualized knee geometry by scaling a template, avoiding the time-consuming tasks of image segmentation, processing, and meshing. These methods could aid physicians in quantifying the current risk of patients developing degenerative musculoskeletal conditions, such as knee OA and in envisioning preventive and conservative measures.

In conclusion, our template-based approach demonstrated promising predictive capabilities for OA development in 97 knees. The method is susceptible to variations in loading, geometry, and threshold for simulating degeneration. Based on our results, the interplay of loading and the criterion for degeneration showed a greater impact on improving the predictions compared to the procedures used to scale cartilage thickness. In addition, the analysis of the lateral compartment should be considered in further developments of our method since it improved the subject stratification by the models into different KL grade groups. Regarding clinical relevance, our method cannot distinguish between future healthy (KL 0–1) and moderate knee OA (KL 2) subjects (AUCs, *p* > 0.05) using information from a radiographically healthy baseline. However, mechanical modeling allows us to identify patients developing severe knee OA (KL 3–4) in the next eight years.

### Supplementary Information

Below is the link to the electronic supplementary material.

Supplementary file1 (PDF 977 kb)
