# Supplementary material for: Knee-Loading Predictions with Neural Networks Improve Finite Element Modeling Classifications of Knee Osteoarthritis: Data from the Osteoarthritis Initiative
Source: Ann Biomed Eng. 2024 Jun 6;52(9):2569–83. doi: 10.1007/s10439-024-03549-2 (PMC11329407; doi:10.1007/s10439-024-03549-2)
Supplement: Supplementary file 1 — Supplementary file1 (PDF 977 kb) [file 10439_2024_3549_MOESM1_ESM.pdf]

Supplementary material for:

Knee-loading predictions with neural networks improve finite element modeling classifications of knee osteoarthritis: data from the Osteoarthritis Initiative

Alexander Paz[1,2], Jere Lavikainen[1], Mikael J. Turunen[1], José J. García[2], Rami K. Korhonen[1], Mika E. Mononen[1]

1 Department of Technical Physics, University of Eastern Finland, Kuopio, Finland

2 Escuela de Ingeniería Civil y Geomática, Universidad del Valle, Cali, Colombia

1. Results

a) Using degeneration thresholds from Mononen et al. 2019.

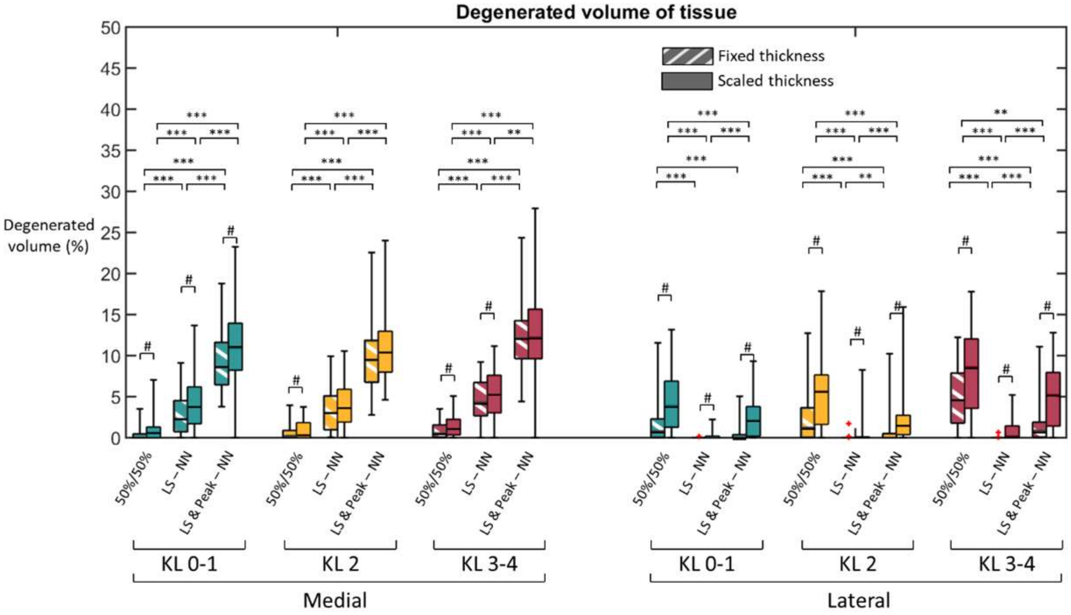

b) Using degeneration thresholds based on Weightman et al. 1978 with N = 10^5 cycles.

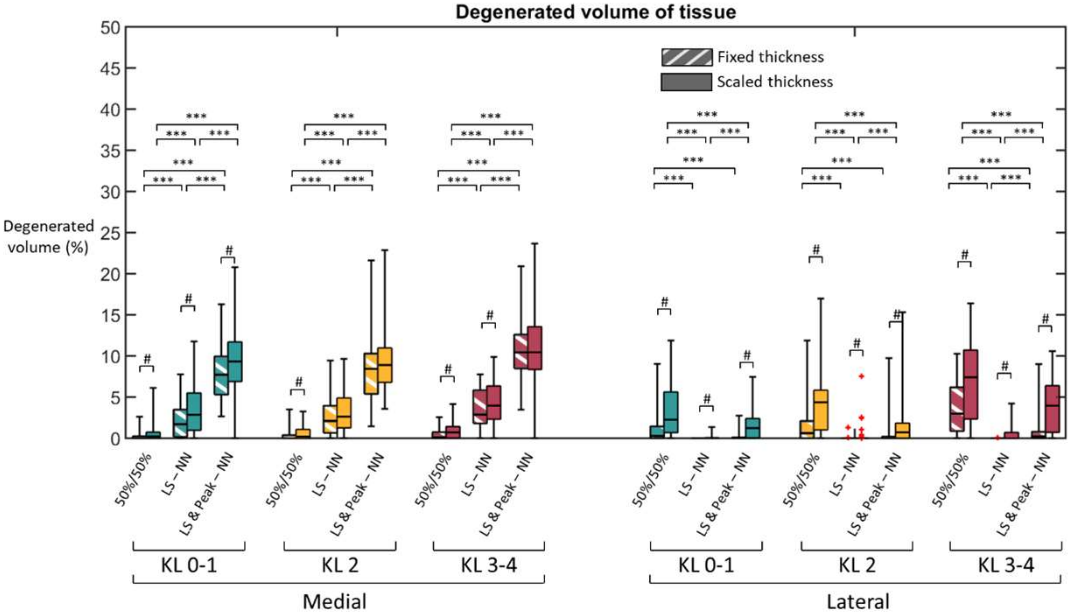

c) Using degeneration thresholds based on Weightman et al. 1978 with N = 10^7 cycles.

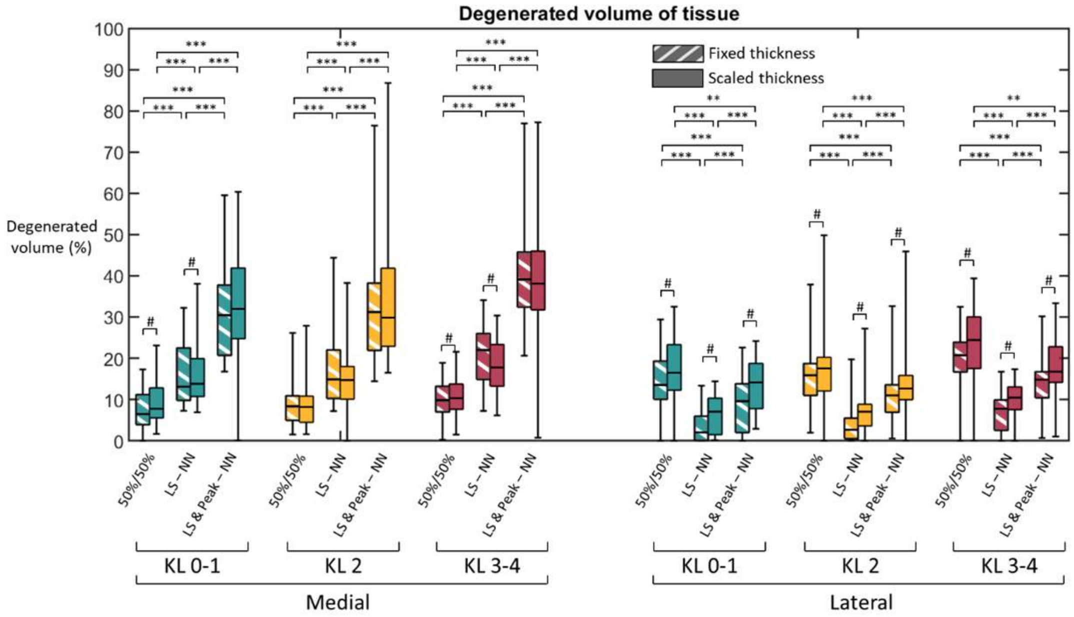

Figure S1. Simulated degenerated percentage volume of tissue for the medial and lateral compartments. Boxes show the first, second, and third quartiles, and whiskers represent the range. 50%/50% – generic evenly distributed joint contact force; LS-NN – load sharing predicted by neural networks with maximums from a generic curve; LS & Peak-NN – load sharing and peak forces predicted by using neural networks. #p < 0.05, *p < 0.05, **p < 0.01, ***p < 0.001.

Classification capabilities

Table S1. Areas under the curve* of logistic ROC analysis, in parenthesis the p values by the DeLong criterion. Analysis was using the failure line proposed by Mononen et al., 2019. Bold font highlights significant AUCs (p<0.05).

**Compartment**	**Lateral**			**Medial**			**Overall**		
**Model**	**KL0-1 vs KL2**	**KL0-1 vs KL3-4**	**KL2 vs KL3-4**	**KL0-1 vs KL2**	**KL0-1 vs KL3-4**	**KL2 vs KL3-4**	**KL0-1 vs KL2**	**KL0-1 vs KL3-4**	**KL2 vs KL3-4**
50% / 50% - Fixed thickness ratio	0.569	0.776	0.705	0.572	0.676	0.606	0.591	0.771	0.704
	(0.699)	**(0.001)**	**(0.010)**	(0.665)	**(0.045)**	(0.330)	(0.568)	**(0.001)**	**(0.016)**
50% / 50% - Scaled thickness ratio	0.554	0.706	0.659	0.515	0.622	0.646	0.532	0.681	0.666
	(0.854)	**(0.016)**	(0.076)	(0.992)	(0.253)	(0.118)	(0.976)	**(0.037)**	(0.067)
LS – NN Fixed thickness ratio	0.524	0.546	0.478	0.568	0.685	0.632	0.565	0.704	0.650
	(0.866)	(0.483)	(0.881)	(0.760)	**(0.032)**	(0.200)	(0.780)	**(0.017)**	(0.109)
LS – NN Scaled thickness ratio	0.454	0.663	0.673	0.505	0.632	0.650	0.495	0.656	0.672
	(0.933)	(0.081)	**(0.012)**	(0.999)	(0.188)	(0.112)	(0.999)	(0.095)	(0.051)
LS & Peaks – NN Fixed thickness ratio	0.533	0.724	0.697	0.536	0.735	0.690	0.535	0.776	0.716
	(0.959)	**(0.005)**	**(0.011)**	(0.931)	**(0.006)**	**(0.019)**	(0.908)	**(0.001)**	**(0.005)**
LS & Peaks – NN Scaled thickness ratio	0.548	0.702	0.713	0.539	0.593	0.629	0.569	0.641	0.706
	(0.943)	**(0.035)**	**(0.004)**	(0.937)	(0.524)	(0.187)	(0.723)	(0.176)	**(0.010)**

*An area of 1.0 indicates perfect differentiation between the groups compared, and an area of 0.5 indicates a random distribution of volumes between the groups. 50%/50% – even load distribution between medial and lateral compartments, LS-NN – load sharing predicted by neural networks, LS & Peaks-NN load sharing, and peak JCF predicted by neural networks.

Table S2. Areas under the curve* of logistic ROC analysis, in parenthesis the p values by the DeLong criterion. Analysis was done using the failure line associated with 10^5 cycles in Eq. 2 (Weightman et al., 1978). Bold font highlights significant AUCs (p<0.05).

**Compartment**	**Lateral**			**Medial**			**Overall**		
**Model**	**KL0-1 vs KL2**	**KL0-1 vs KL3-4**	**KL2 vs KL3-4**	**KL0-1 vs KL2**	**KL0-1 vs KL3-4**	**KL2 vs KL3-4**	**KL0-1 vs KL2**	**KL0-1 vs KL3-4**	**KL2 vs KL3-4**
50% / 50% - Fixed thickness ratio	0.574	0.775	0.699	0.534	0.605	0.572	0.582	0.768	0.696
	(0.649)	**(0.001)**	**(0.013)**	(0.940)	(0.328)	(0.607)	(0.614)	**(0.001)**	**(0.017)**
50% / 50% - Scaled thickness ratio	0.549	0.704	0.657	0.556	0.593	0.640	0.536	0.683	0.664
	(0.877)	**(0.019)**	(0.077)	(0.837)	(0.528)	(0.113)	(0.963)	**(0.034)**	(0.073)
LS – NN Fixed thickness ratio	0.527	0.516	0.512	0.581	0.684	0.619	0.581	0.699	0.636
	(0.499)	(0.861)	(0.930)	(0.647)	**(0.033)**	(0.284)	(0.650)	**(0.018)**	(0.181)
LS – NN Scaled thickness ratio	0.479	0.642	0.638	0.512	0.625	0.650	0.493	0.648	0.664
	(0.994)	(0.096)	**(0.038)**	(0.994)	(0.229)	(0.111)	(0.999)	(0.125)	(0.067)
LS & Peaks – NN Fixed thickness ratio	0.503	0.701	0.685	0.541	0.733	0.689	0.545	0.772	0.713
	(0.999)	**(0.012)**	**(0.010)**	(0.916)	**(0.006)**	**(0.021)**	(0.868)	**(0.001)**	**(0.006)**
LS & Peaks – NN Scaled thickness ratio	0.437	0.697	0.715	0.548	0.589	0.636	0.580	0.657	0.717
	(0.873)	**(0.047)**	**(0.004)**	(0.882)	(0.558)	(0.154)	(0.671)	(0.132)	**(0.005)**

*An area of 1.0 indicates perfect differentiation between the groups compared, and an area of 0.5 indicates a random distribution of volumes between the groups. 50%/50% – even load distribution between medial and lateral compartments, LS-NN – load sharing predicted by neural networks, LS & Peaks-NN load sharing, and peak JCF predicted by neural networks.

Table S3. Areas under the curve* of logistic ROC analysis, in parenthesis the p values by the DeLong criterion. Analysis was done using the failure line associated with 10^7 cycles in Eq. 2 (Weightman et al., 1978). Bold font highlights significant AUCs (p<0.05).

**Compartment**	**Lateral**			**Medial**			**Overall**		
**Model**	**KL0-1 vs KL2**	**KL0-1 vs KL3-4**	**KL2 vs KL3-4**	**KL0-1 vs KL2**	**KL0-1 vs KL3-4**	**KL2 vs KL3-4**	**KL0-1 vs KL2**	**KL0-1 vs KL3-4**	**KL2 vs KL3-4**
50% / 50% Fixed thickness ratio	0.541	0.728	0.707	0.553	0.667	0.636	0.543	0.709	0.689
	(0.951)	**(0.005)**	**(0.013)**	(0.891)	(0.057)	(0.188)	(0.945)	**(0.011)**	**(0.030)**
50% / 50% Scaled thickness ratio	0.514	0.706	0.691	0.499	0.653	0.666	0.502	0.689	0.696
	(0.996)	**(0.018)**	**(0.021)**	(1.000)	(0.100)	(0.065)	(1.000)	**(0.031)**	**(0.019)**
LS – NN Fixed thickness ratio	0.541	0.725	0.701	0.549	0.690	0.659	0.544	0.714	0.693
	(0.945)	**(0.006)**	**(0.017)**	(0.909)	**(0.026)**	(0.086)	(0.940)	**(0.009)**	**(0.025)**
LS – NN Scaled thickness ratio	0.488	0.706	0.717	0.515	0.615	0.646	0.500	0.653	0.682
	(0.998)	**(0.018)**	**(0.006)**	(0.989)	(0.286)	(0.130)	(0.999)	(0.084)	**(0.042)**
LS & Peaks – NN Fixed thickness ratio	0.569	0.715	0.685	0.520	0.731	0.723	0.556	0.749	0.714
	(0.815)	**(0.006)**	**(0.042)**	(0.994)	**(0.005)**	**(0.006)**	(0.869)	**(0.002)**	**(0.010)**
LS & Peaks – NN Scaled thickness ratio	0.444	0.670	0.725	0.468	0.623	0.662	0.460	0.650	0.694
	(0.822)	(0.071)	**(0.004)**	(0.951)	(0.257)	(0.070)	(0.919)	(0.125)	**(0.019)**

*An area of 1.0 indicates perfect differentiation between the groups compared, and an area of 0.5 indicates a random distribution of volumes between the groups. 50%/50% – even load distribution between medial and lateral compartments, LS-NN – load sharing predicted by neural networks, LS & Peaks-NN load sharing, and peak JCF predicted by neural networks.

2. References

Mononen, M.E., Liukkonen, M.K., Korhonen, R.K., 2019. Utilizing Atlas-Based Modeling to Predict Knee Joint Cartilage Degeneration: Data from the Osteoarthritis Initiative. Ann. Biomed. Eng. 47, 813–825. https://doi.org/10.1007/s10439-018-02184-y

Weightman, B., Chappell, D.J., Jenkins, E.A., 1978. A second study of tensile fatigue properties of human articular cartilage. Ann. Rheum. Dis. 37, 58–63. https://doi.org/10.1136/ard.37.1.58
